# Visualization of Exo- and Endocytosis of AMPA Receptors During Hippocampal Synaptic Plasticity Around Postsynaptic-Like Membrane Formed on Glass Surface

**DOI:** 10.3389/fncel.2018.00442

**Published:** 2018-11-21

**Authors:** Tomoo Hirano

**Affiliations:** Department of Biophysics, Graduate School of Science, Kyoto University, Kyoto, Japan

**Keywords:** exocytosis, endocytosis, LTP, LTD, hippocampus, AMPA receptor, total internal reflection fluorescence microscopy, live-cell imaging

## Abstract

Regulation of exo- and endocytosis of α-amino-3-hydroxy-5-methyl-4-isoxazolepropionic acid (AMPA)-type glutamate receptor (AMPAR) plays a critical role in the expression of synaptic plasticity such as long-term potentiation (LTP) and long-term depression (LTD) at excitatory central synapses. Enhanced AMPAR exocytosis or endocytosis has been suggested to contribute to LTP or LTD, respectively. However, several unsettled fundamental questions have remained about AMPAR exo- and endocytosis in the basal condition and during synaptic plasticity: (1) Does the size of each exo- or endocytosis event, and/or do the frequencies of these events change during LTP or LTD? If they change, what are the time courses of the respective changes? (2) Where does the exo- or endocytosis preferentially occur in each condition: inside or in the vicinity of postsynaptic membrane, or in the extrasynaptic membrane? (3) Do different types of AMPAR, such as GluA1 homo-tetramer, GluA1/2 hetero-tetramer and GluA2/3 hetero-tetramer, show distinct exo- and endocytosis changes? To address these questions, we developed new methods to observe individual events of AMPAR exo- or endocytosis with a high signal to noise (SN) ratio in a culture preparation using total internal reflection fluorescence microscopy (TIRFM). In these studies, hippocampal neurons were cultured on a neurexin (NRX)-coated glass coverslip, which induced formation of postsynaptic-like membrane (PSLM) directly on the glass surface. Then, a super-ecliptic pHluorin (SEP)-tagged AMPAR subunit such as GluA1 (GluA1-SEP) was expressed in neurons and its fluorescence changes during LTP induced by high frequency electrical field stimulation were observed with TIRFM, which showed different time courses of exocytosis changes of GluA1-, GluA2-, or GluA3-SEP in and around PSLM. In addition, a new method to detect individual endocytosis events of AMPAR was developed by combining TIFRM observation of GluA-SEP around PSLM with a rapid extracellular pH exchange method using a U-tube. Recent results on exo- and endocytosis changes of GluA-SEP during N-methyl-D-aspartate (NMDA)-induced LTD suggested that suppression of AMPAR exocytosis rather than enhancement of AMPAR endocytosis primarily contributes to LTD expression, although the NMDA application transiently enhances clathrin-dependent endocytosis of GluA1-containing AMPAR.

## Introduction

Long-term potentiation (LTP) and long-term depression (LTD) at hippocampal glutamatergic synapses have been regarded as basic cellular mechanisms of learning and memory, and intensively studied (Malinow and Malenka, 2002; Kauer and Malenka, 2007; Collingridge et al., 2010; Huganir and Nicoll, 2013). Originally changes in functional properties of α-amino-3-hydroxy-5-methyl-4-isoxazolepropionic acid (AMPA)-type glutamate receptor (AMPAR) were considered as molecular mechanisms of LTP or LTD. More recently, changes in the number of AMPAR on the postsynaptic membrane attracted much attention of synaptic physiologists as primary mechanisms of LTP or LTD expression (Malinow and Malenka, 2002; Kennedy and Ehlers, 2006; Derkach et al., 2007; Shepherd and Huganir, 2007; Huganir and Nicoll, 2013). AMPAR exocytosis, endocytosis, lateral movement on the plasma membrane and trapping in the postsynaptic membrane regulate the number of postsynaptic AMPARs (Malinow and Malenka, 2002; Shepherd and Huganir, 2007; Makino and Malinow, 2009; Opazo and Choquet, 2011; Huganir and Nicoll, 2013; Wu et al., 2017).

Enhancement of AMPAR exocytosis was suggested to contribute to LTP expression (Shi et al., 1999; Hayashi et al., 2000; Passafaro et al., 2001; Kopec et al., 2006; Park et al., 2006; Plant et al., 2006; Lin et al., 2009; Kennedy et al., 2010; Patterson et al., 2010; Huganir and Nicoll, 2013). Exocytosis was reported to occur around the postsynaptic membrane and in extra-synaptic membrane away from synapses (Kennedy et al., 2010). In the latter case, lateral movement of AMPAR and trapping of it on the postsynaptic membrane are necessary to accumulate AMPAR in the postsynaptic membrane (Opazo et al., 2010, 2012; Opazo and Choquet, 2011; Chen et al., 2015). However, the extent to which each pathway contributes to LTP expression remains an open question.

There are four types of AMPAR subunits GluA1–4 (Hollmann and Heinemann, 1994; Dingledine et al., 1999). In hippocampal glutamatergic synapses GluA1/GluA2 hetero-tetramer and GluA2/GluA3 hetero-tetramer are the main postsynaptic receptors (Dingledine et al., 1999). On the other hand, GluA1 homo-tetramer is present in some types of hippocampal neurons, and its involvement in synaptic plasticity has also been reported (Iino et al., 1990; Plant et al., 2006; Lu Y. et al., 2007; Sanderson et al., 2016). Thus, there may be specific regulatory mechanisms for each of these subtypes of AMPAR composed of different combinations of subunits, but this has not yet been precisely clarified.

Enhancement of AMPAR endocytosis has been suggested as a primary mechanism for LTD expression (Beattie et al., 2000; Lee et al., 2002; Ashby et al., 2004; Lin and Huganir, 2007; Fernández-Monreal et al., 2012). Both clathrin-dependent and -independent AMPAR endocytosis occur, and the contribution of the former to LTD induction has been reported (Glebov et al., 2015; Zheng et al., 2015). AMPAR endocytosis might occur not only in the extrasynaptic membrane but also in the vicinity of postsynaptic membrane (Blanpied et al., 2002; Kennedy and Ehlers, 2006; Lu J. et al., 2007; Tao-Cheng et al., 2011; Fujii et al., 2017, 2018). The involvement of GluA1 homo-tetramer in LTD induction has been suggested (Sanderson et al., 2016), although involvement of GluA2-lacking AMPAR in LTP or LTD has been debated (Passafaro et al., 2001; Plant et al., 2006; Adesnik and Nicoll, 2007; Gray et al., 2007; Lu Y. et al., 2007). AMPAR subtype changes might also take place during LTD.

LTP and LTD have been studied mainly by electrophysiological recording, immuno-cytological staining and biochemical assays combined with pharmacological or molecular biological manipulations and/or use of transgenic mice (Malinow and Malenka, 2002; Kauer and Malenka, 2007; Collingridge et al., 2010; Huganir and Nicoll, 2013). The use of live-cell imaging techniques in the analyses of LTP and LTD mechanisms has increased. Technical advancements have made it possible to detect individual events of exo- or endocytosis of AMPAR. Development of a pH-sensitive variant of green fluorescent protein called super-ecliptic pHluorin (SEP) enabled selective monitoring of proteins in neutral pH conditions, such as on the cell-surface, but not proteins inside intracellular organelles with acidic luminal solution (Miesenböck et al., 1998). SEP has been widely used in studies on AMPAR trafficking during LTP or LTD (Ashby et al., 2004; Lin and Huganir, 2007; Yudowski et al., 2007; Lin et al., 2009; Araki et al., 2010; Kennedy et al., 2010; Tanaka and Hirano, 2012; Rathje et al., 2013; Jullié et al., 2014; Tanaka et al., 2014; Fujii et al., 2017, 2018; Rosendale et al., 2017; Temkin et al., 2017; Wu et al., 2017). Total internal reflection fluorescence microscopy (TIRFM) provides very high signal/noise (SN) ratio images by limiting the depth of the visualization zone (Axelrod, 2001), and has also been used in live-cell imaging studies of AMPAR dynamics (Yudowski et al., 2007; Wang et al., 2008; Lin et al., 2009; Araki et al., 2010; Tanaka and Hirano, 2012; Jullié et al., 2014; Tanaka et al., 2014; Fujii et al., 2017, 2018; Rosendale et al., 2017). A rapid extracellular pH exchange method combined with the use of SEP made it possible to record individual endocytosed vesicles (Merrifield et al., 2005; Jullié et al., 2014; Rosendale et al., 2017).

Recently, we developed a new method to further improve the SN ratio and spatiotemporal resolution of live-cell imaging data of SEP-tagged AMPAR. We induced formation of postsynaptic-like membrane (PSLM) directly on the surface of a glass coverslip, and then studied the dynamics of GluA-SEP around PSLM during LTP or LTD expression (Tanaka and Hirano, 2012; Tanaka et al., 2014; Fujii et al., 2017). In this review, I will briefly summarize recent results on AMPAR dynamics during synaptic plasticity obtained using GluA-SEP, PSLM and TIRFM.

## Formation of PSLM on Neurexin-Coated Glass

Several types of cell-adhesion molecules are found at synapses such as Neuroligin (NLG), Neurexin (NRX), Synaptic cell adhesion molecule (SynCAM), EphrinB, leucine rich repeat transmembrane (LRRTM) and N-Cadherin. Among them presynaptic membrane protein NRX and postsynaptic membrane protein NLG have been studied extensively (Levinson and El-Husseini, 2005; Dean and Dresbach, 2006; Craig and Kang, 2007; Südhof, 2008; Bukalo and Dityatev, 2012). Both of them have different subtypes and various splice variants. There are five NLG genes NLG 1–4 and NLG 4Y, and there are six NRX genes NRX 1α, 1β, 2α, 2β, 3α, 3β. NRXs undergo extensive alternative splicing, which could potentially generate >2,000 variants. Among these variants, splicing insertion of site 4 in β-NRX promotes GABAergic synapse formation, whereas β-NRX without site 4 insertion promotes glutamatergic synapse formation. It is also known that NLG 1 with splice insertion at site B promotes glutamatergic synapse formation, and that NLG 2 is primarily found at GABAergic synapses.

**Figure 1:**
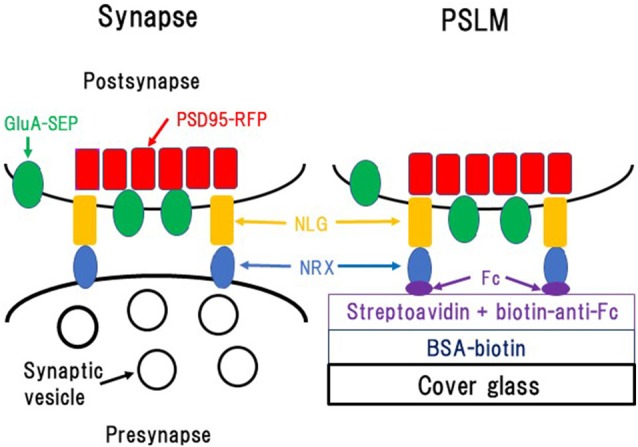
Formation of postsynaptic-like membrane (PSLM). NRX, Neurexin; NLG, Neuroligin; BSA, Bovine serum albumin; Fc, fragment crystallizable of immunoglobulin. This figure is newly drawn based on our previous publications (Tanaka and Hirano, 2012; Tanaka et al., 2014), and copyright permission is not required.

**Figure 2:**
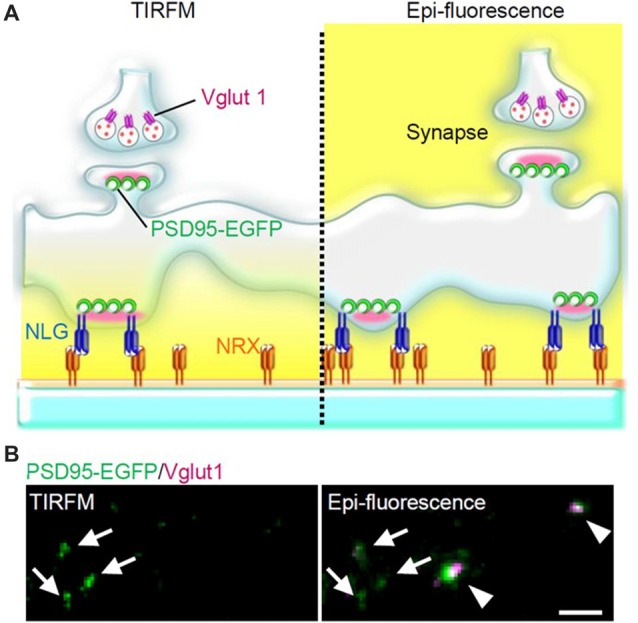
PSLM and normal synapses observed with total internal reflection fluorescence microscopy (TIRFM) or with conventional epi-fluorescence. **(A)** Scheme of PSLM and a normal synapse on NRX-coated glass. Excitation light (yellow) reaches only PSLM and the lower parts of dendrites in TIRFM (left), whereas it covers the whole area under epi-fluorescence (right). At a normal synapse, postsynaptic PSD95 signal is apposed to presynaptic vglut1 signal. **(B)** PSD95-EGFP signal (green) and vglut1 signal (magenta) recorded with TIRFM (left) or with epi-fluorescence (right), respectively. Arrows indicate PSLMs that are clearly observed with TIRFM and are not accompanied by vglut1 signals, and arrowheads indicate normal synapses which are not clearly observed with TIRFM. These figure panels were first published in Tanaka et al. (2014), and copyright permission was obtained.

NLG expressed in non-neuronal cells co-cultured with neurons induces formation of presynaptic structures in axons, while NRX when similarly expressed induces formation of postsynaptic structures in dendrites (Scheiffele et al., 2000; Graf et al., 2004). Furthermore, NRX attached to beads induces clustering of postsynaptic proteins (Graf et al., 2004). These findings prompted us to test whether a glass coverslip coated with NRX could induce formation of postsynaptic structures on the glass surface. We considered that such postsynaptic structures formed directly on and parallel to the glass surface would be an ideal model of postsynaptic structure which could be used in live-cell fluorescence imaging experiments using TIRFM (Figures 1, 2), because application of TIRFM to such structures would be efficient and effective. TIRFM can provide very high contrast fluorescence images by decreasing background signals. Excitation light reaches only about 100 nm above the glass surface in an inverted microscope equipped for TIRFM (Figure 2).

Glass coating with NRX was performed utilizing biotin-avidin interaction and an antibody which was described in detail elsewhere (Tanaka and Hirano, 2012; Tanaka et al., 2014; Figure 1). Briefly, glass coverslips were coated with bovine serum albumin (BSA) conjugated with biotin. Then, streptavidin, which binds to biotin, was overlaid. Next, the anti-fragment crystallizable (Fc) region of human immunoglobulin conjugated to biotin, which binds to streptavidin, was applied. Finally, NRX 1β without splicing insertion at site 4 fused to the FC region of human immunoglobulin was applied so that it was captured by the anti-Fc antibody.

When hippocampal neurons overexpressing NLG 1 with splice insertion at site B were cultured on the NRX-coated coverslip, many punctate structures that contained PSD95, a marker protein of postsynaptic density, but that were not apposed to a presynaptic marker were observed with TIRFM (Figure 2). Such postsynaptic-like membrane (PSLM) exhibited accumulation of homer, another marker protein of postsynaptic density and AMPAR. We chose relatively large neurons possessing spines which presumably corresponded to pyramidal neurons for the experiments. Conditioning stimulation inducing either LTP or LTD increased or decreased the amount of AMPAR in PSLM, respectively (Tanaka and Hirano, 2012; Fujii et al., 2018).

## Exocytosis Regulation in LTP Around PSLM

**Figure 3:**
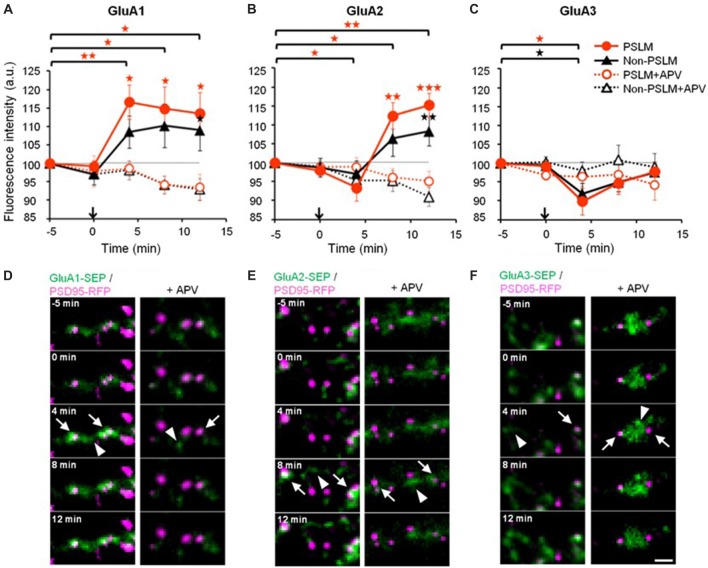
Changes of α-amino-3-hydroxy-5-methyl-4-isoxazolepropionic acid (AMPA)-type glutamate receptor (AMPAR) subunit number by long-term potentiation (LTP)-inducing stimulation. **(A–C)** Averaged time courses of GluA1–3 fluorescence intensity in PSLM (red) and in non-PSLM (black) measured every 4 min before and after the field stimulation (arrows). Data in the presence of APV (+APV) are also shown (dotted lines). Error bars indicate SEM. *p < 0.05, **p < 0.01 and ***p < 0.001. **(D–F)** GluA-super-ecliptic phluorin (SEP) signals (green) and PSD95-RFP signal (magenta) are shown. PSD95-RFP was recorded before the stimulation, and images of the two signals were overlaid. GluA-SEP signals in PSLM and non-PSLM are indicated by arrows and arrowheads, respectively. Scale bar, 2 μm. These figure panels were first published in Tanaka and Hirano (2012), and copyright permission is not necessary.

We reported that high frequency electrical field stimulation used to induce LTP increases the amount of SEP-tagged GluA subunit of AMPAR in and outside PSLM in a hippocampal culture preparation (Figure 3, Tanaka and Hirano, 2012). The increase is somewhat larger in PSLM. By the way, PSLM is not apposed to a presynaptic terminal releasing glutamate. We considered that glutamate released from nearby presynaptic terminals activates N-methyl-D-aspartate (NMDA) receptors and induces the changes in the amount of GluA-SEP in PSLM, because an antagonist of NMDA receptor APV suppresses the changes.

**Figure 4:**
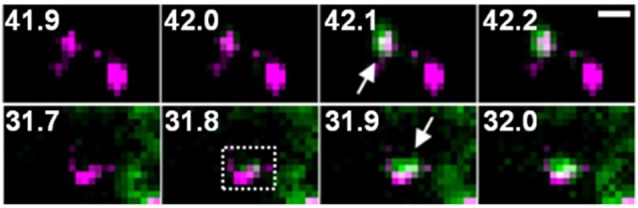
Two examples of GluA1-SEP (green) exocytosis (arrows) shown together with PSD95-RFP (magenta). The numbers indicate time (seconds) after the field stimulation. These figure panels were first published in Tanaka and Hirano (2012), and copyright permission is not necessary.

One possible factor contributing to the increase in the amount of GluA-SEP on the surface is enhancement of GluA-SEP exocytosis. Individual events of GluA-SEP exocytosis can be observed around PSLM by high frequency live-cell TIRFM imaging (Figure 4). LTP-inducing electrical stimulation increases the frequency of GluA-SEP exocytosis. We reported a transient (about 1 min) increase of GluA1-SEP exocytosis frequency around PSLM and a subsequent increase for several minutes outside PSLM. We also found that GluA1-SEP exocytosis does not occur in the center of PSLM, but rather it occurs in the periphery of PSLM or outside of PSLM. Exocytic domain adjacent to the postsynaptic membrane was previously reported (Kennedy et al., 2010).

**Figure 5:**
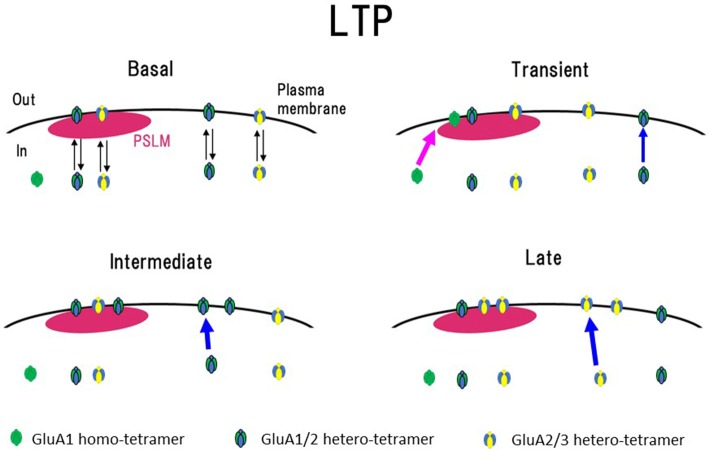
Scheme of exocytosis changes of each AMPAR subtype during LTP expression. In a basal condition (Basal), exo- and endocytosis of GluA1/GluA2 and GluA2/GluA3 hetero-tetramer are in equilibrium. Soon after the high frequency electrical stimulation (Transient, around 1 min after the stimulation), exocytosis of GluA1 homo-tetramer occurs in the periphery of PSLM (red arrow), and exocytosis of GluA1/GluA2 increases outside PSLM (blue arrow). In the following period (Intermediate, about 3–10 min after the stimulation), exocytosis of GluA1/GluA2 increases outside PSLM (blue arrow). Finally (Late, about 20 min after the stimulation) exocytosis of GluA2/GluA3 increases outside PSLM (blue arrow). Some AMPAR exocytosed outside PSLM are likely to move into PSLM by lateral diffusion on the plasma membrane. This figure is newly drawn based on our previous publication (Tanaka and Hirano, 2012), and copyright permission is not required.

Changes in the cell surface amounts and in the exocytosis frequencies of GluA2-SEP or GluA3-SEP also occur during LTP expression (Tanaka and Hirano, 2012). GluA2-SEP and GluA3-SEP show different time courses of these changes (Figure 3). Co-expression experiments of GluA1-SEP/GluA2, GluA1/GluA2-SEP or GluA2/GluA3-SEP were also performed. Based on the experimental results, we proposed the following scheme as a mechanism of the expression of LTP (Figure 5). (1) Exocytosis of GluA1 homo-tetramer occurs particularly in the periphery of PSLM immediately after the conditioning stimulation. (2) A few minutes after the conditioning stimulation, exocytosis of GluA1/GluA2 hetero-tetramer increases for several minutes outside PSLM. Some of the exocytosed GluA1/GluA2 hetero-tetramers may move into PSLM by diffusion on the plasma membrane. (3) From about 20 min after the conditioning stimulation exocytosis of GluA2/GluA3 gradually increases outside PSLM. The above scheme suggests that changes in the distribution of AMPAR subtypes are likely to occur during LTP. I presume that exo- and endocytosis of GluA1/GluA2 and GluA2/GluA3 hetero-tetramers are in equilibrium in a basal condition.

Regarding changes of AMPAR exocytosis during LTP expression, some unclear or conflicting observations have been reported about sites of exocytosis, AMPAR subtype specificity and precise time courses. Some previous studies reported different molecular regulation mechanisms between constitutive AMPAR exocytosis and regulated exocytosis during LTP induction (Ahmad et al., 2012; Temkin et al., 2017; Wu et al., 2017). Yudowski et al. (2007), Lin et al. (2009) and Makino and Malinow (2009) reported that the majority of GluA1 exocytosis occurred in extrasynaptic membrane, whereas Kennedy et al. (2010) found exocytic domains adjacent to postsynaptic density. The involvement of GluA2-lacking AMPAR such as GluA1 homo-tetramer in LTP has also been controversial (Passafaro et al., 2001; Plant et al., 2006; Adesnik and Nicoll, 2007; Gray et al., 2007; Lu Y. et al., 2007). Furthermore, there has been little precise information about how exocytosis of each subtype of AMPAR, such as GluA1/2 or GluA2/3 hetero-tetramer changes during LTP expression. Tanaka and Hirano (2012) provided some answers or information regarding these questions. However, I would like to note the following. First, PSLM is an artificial structure and may not necessarily express all normal functions of hippocampal glutamatergic postsynaptic membrane. Second, over-expressed GluA1-SEP might affect normal cellular processes. These points will be discussed later.

## Endo-and Exocytosis Regulation During LTD Around PSLM

**Figure 6:**
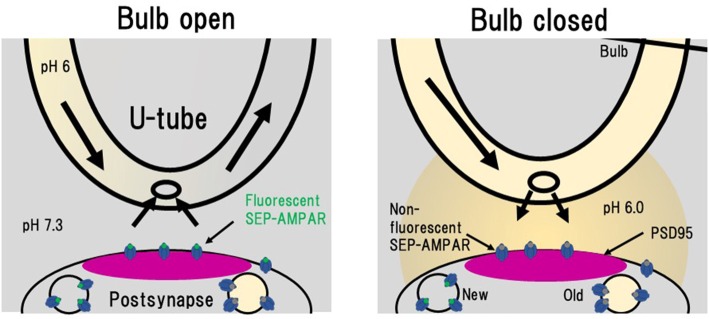
Rapid extracellular pH exchange method using a U-tube and detection of individual AMPAR endocytosis. When a bulb on a U-tube is open (Bulb open), the pH 6.0 solution flows inside U-tube and the extracellular pH 7.3 solution is also soaked into the U-tube. When the bulb is closed (Bulb closed), the intra U-tube pH 6.0 solution leaks out to the extracellular solution. In this pH 6.0 condition, only SEP signals from intracellular vesicles with near-neutral luminal pH such as those immediately after endocytosis can be detected. This figure is newly drawn based on our previous publication (Fujii et al., 2017), and copyright permission is not required.

NMDA application induces LTD in hippocampal culture preparations, which is accompanied by a decrease in amount of cell-surface AMPAR (Lee et al., 1998; Beattie et al., 2000; Collingridge et al., 2010; Fernández-Monreal et al., 2012). Enhancement of clathrin-dependent endocytosis has been considered to contribute to LTD expression (Glebov et al., 2015; Zheng et al., 2015). In order to obtain better understanding of the process of LTD expression, Fujii et al. (2017, 2018) used PSLM for analyses of AMPAR endocytosis during LTD. Individual endocytic events of cell surface molecules, including AMPAR, have been detected by the combination of a rapid extracellular pH change method with the use of SEP (Jullié et al., 2014; Rosendale et al., 2017). The extracellular pH change was performed with a θ tube in these studies. We used a U-tube instead of a θ tube for the extracellular pH exchange (Fujii et al., 2017, 2018; Figure 6). The use of a U-tube enables us to remove the applied solution from the experimental chamber, although the speed of solution exchange is somewhat slower than that of a method using a θ tube. Using the combination of rapid extracellular pH change and GluA1-SEP, individual events of GluA1-SEP endocytosis were observed around PSLM.

This combination method is also useful to precisely determine the amount of cell surface AMPAR, because some SEP fluorescence arises from molecules located in endoplasmic reticulum with relatively neutral luminal pH (Paroutis et al., 2004; Rathje et al., 2013). By subtracting fluorescence signals at pH 5.5 from those at pH 7.3, cell-surface signals can be isolated. This method allowed us to precisely analyze cell-surface amounts of GluA1-SEP and GluA2-SEP during LTD expression (Fujii et al., 2018). The thus estimated cell-surface amounts of both GluA1-SEP and GluA2-SEP gradually decrease after the NMDA application. The decrease of GluA1-SEP is sustained for more than 30 min, while that of GluA2-SEP tends to recover (Fujii et al., 2018).

The LTD-inducing NMDA application transiently increases the size of individual GluA1-SEP endocytic events and also the frequency of GluA1-SEP endocytosis for about 1 min. Interestingly this transiently enhanced large endocytosis is clathrin-dependent, whereas constitutive endocytosis of GluA1-SEP does not depend on clathrin (Fujii et al., 2017, 2018). This result is consistent with a previous study reporting that basal endocytosis of AMPAR does not depend on clathrin, but that the NMDA-induced endocytosis depends on clathrin (Glebov et al., 2015). Thus, there are at least two independent endocytosis pathways for AMPAR. We also reported that clathrin-dependent GluA1-SEP endocytosis induced by the NMDA application preferentially takes place in the periphery of PSLM, which is likely to correspond to the endocytic zone adjacent to the postsynaptic membrane (Blanpied et al., 2002; Lu J. et al., 2007).

**Figure 7:**
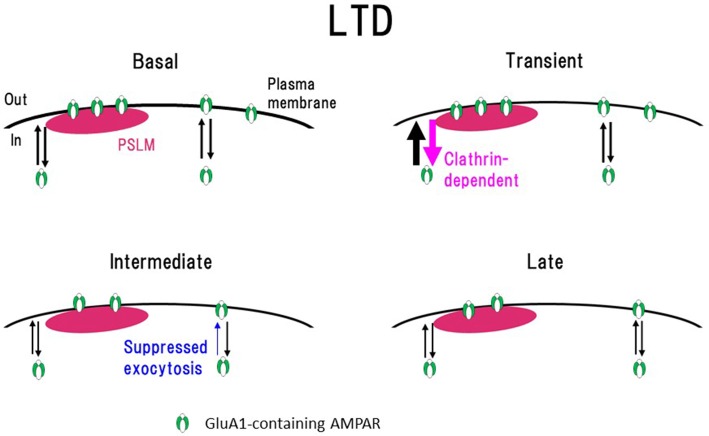
Scheme of exo- and endocytosis changes of GluA1-containing AMPAR during long-term depression (LTD) expression. In a basal condition (Basal) exo- and endocytosis of GluA1-containing AMPAR are in equilibrium. Immediately after the N-methyl-D-aspartate (NMDA) application (Transient, around 1 min after the onset of NMDA application), clathrin-dependent endocytosis and exocytosis of AMPAR increase in the periphery of PSLM. In the following period (Intermediate, about 3–10 min), AMPAR exocytosis is suppressed. Finally (Late) exo- and endocytosis of AMPAR go into equilibrium in a low level. How each type of AMPAR changes during LTD remains to be clarified. This figure is newly drawn based on our previous publications (Fujii et al., 2017, 2018), and copyright permission is not required.

Transient enhancement of GluA1-SEP endocytosis seems to be insufficient to explain slowly developing LTD expression. Considering that the cell-surface amounts of molecules are regulated by the balance of endo- and exocytosis, we examined changes of GluA1-SEP exocytosis after the LTD-inducing NMDA application (Fujii et al., 2018). We found that after the NMDA application, GluA1-SEP exocytosis is transiently enhanced and then decreased. Taking all these results together, it was suggested that sustained suppression of AMPAR exocytosis, rather than enhanced endocytosis, plays a predominant role in LTD expression (Figure 7).

In addition, the cell-surface amount, and endo- and exocytosis of GluA2-SEP after the NMDA application were examined. We showed that GluA2-SEP exhibits different temporal profiles from those of GluA1-SEP, suggesting differential regulation of GluA2-SEP. However, how each type of AMPAR consisting of GluA1–3, such as GluA1/2 or GluA2/3 hetero-tetramer, changes during LTD has not been reported. Transient and simultaneous enhancement of both exo- and endocytosis of GluA1 after the onset of NMDA application might contribute to substitutions of AMPAR subtypes. GluA2-containing AMPAR might be replaced by Ca^2+^ permeable AMPAR lacking GluA2 (Sanderson et al., 2016).

Importantly, similar changes of GluA1-SEP dynamics during LTD expression were observed in conventional synapses. Using oblique illumination, we studied the exo- and endocytosis changes of GluA1-SEP around conventional synapses in hippocampal neurons cultured on glass that was not coated with NRX (Fujii et al., 2018). Although, the SN ratio of fluorescence images of GluA1-SEP around synapses observed with oblique illumination was inferior to that obtained using PSLM and TIRFM, qualitatively similar results were obtained.

## Lateral Movement of AMPAR on the Plasma Membrane

AMPAR moves around on the plasma membrane by lateral diffusion, and this movement has been studied by live-cell imaging of a fluorescent quantum dot attached to AMPAR (Bats et al., 2007; Groc et al., 2008). Diffusion is much faster in extrasynaptic membrane than in postsynaptic membrane. Notably, there is little movement of AMPAR in the postsynaptic membrane for a long time. Thus, AMPAR can be trapped at a postsynaptic membrane. Together with the balance of exo- and endocytosis, the efficiency of trapping of AMPAR at a postsynaptic membrane or the balance of coming-in and going-out of AMPAR to and from a postsynaptic membrane should influence the amount of AMPAR at a postsynaptic membrane (Opazo et al., 2010, 2012; Opazo and Choquet, 2011; Chen et al., 2015).

## Merits and Demerits of PSLM

High SN ratio images of fluorescent molecules can be obtained around PSLM with TIRFM through the reduction of background signals (Figure 2). Parallel formation of PSLM on the glass surface enables a simple interpretation of imaging data about how synaptic proteins are localized in and around PSLM. PSLM is also stable and does not move, whereas dendritic spines in which postsynaptic membrane is located occasionally move in culture and *in vivo* (Deng and Dunaevsky, 2005). In addition, PSLM can be found much more easily under TIRFM than conventional postsynaptic membranes (Tanaka et al., 2014). These points are significant merits of using PSLM for analyses of postsynaptic processes in a basal condition and during synaptic plasticity.

However, PSLM is certainly an artificial structure deficient in interaction with presynaptic structures, which could potentially affect some functions of postsynaptic membrane. Thus, certain care should be taken in interpretation of results obtained using PSLM. Nevertheless, PSLM retains essential properties of postsynaptic membrane, as evidenced by the accumulation of postsynaptic proteins such as PSD95 and homer, and dynamic changes of the amount of AMPAR relevant to the expression of LTP and LTD. Furthermore, it was demonstrated that exo- and endocytic changes of GluA1-SEP during LTD at conventional postsynaptic membranes were qualitatively similar to those observed at PSLM as explained above Fujii et al. (2018). Thus, PSLM can be regarded as a useful experimental model and can provide guiding results and/or ideas that would be worth rigorously examining at conventional synapses.

## Future Directions

There are many different types of synapses. Some are excitatory, and the others are inhibitory. There are also large differences in presynaptic transmitter release probability among synapses, which is likely to affect postsynaptic properties (Konnerth et al., 1990; Miyawaki and Hirano, 2011; Biederer et al., 2017). Synapses between a pair of neurons often change their characteristics during development (Pouzat and Hestrin, 1997; Yu and Goodrich, 2014). There are many types of synaptic adhesion molecules such as NLG, NRX, SynCAM, EphrinB, LRRTM and N-cadherin. In addition, there are different subtypes and different splice variants of NRX and NLG as explained above. They are differently distributed among synapses, and some of them are co-localized at a synapse. Combination of pre- and postsynaptic adhesion molecules are likely to play critical roles in determination of synaptic properties (Levinson and El-Husseini, 2005; Dean and Dresbach, 2006; Craig and Kang, 2007; Südhof, 2008; Bukalo and Dityatev, 2012). Changing a glass-coating presynaptic adhesion molecule, adding another coating molecule, or changing cultured neuronal type might reveal synapse-type specific postsynaptic properties. Such experiments might also provide useful information about determinant molecules for functional properties of a particular type of synapse.

Over-expression of AMPAR-SEP is also artificial manipulation which could affect normal cellular processes. Overexpression of a subunit of AMPAR such as GluA1 inevitably increases its relative amount, potentially affecting normal cellular processes. Indeed, we found that GluA2-SEP behaves somewhat differently depending on whether it is co-expressed with GluA1 (Tanaka and Hirano, 2012). I also note that SEP is not small in size and could affect AMPAR functions and regulations. One way to overcome these problems is to label endogenous AMPAR with a small fluorescent molecular probe (Wakayama et al., 2017).

Development of new fluorescent proteins such as pH-sensitive red fluorescent proteins will enable us to simultaneously monitor multiple proteins, and will certainly promote the analyses (Shen et al., 2014; Martineau et al., 2017). I would also like to note that application of super-resolution fluorescence imaging techniques, Stochastic optical reconstruction microscopy (STORM) and Photo-activated localization microscopy (PALM) in particular, were likely to match very well with analyses on PSLM, which is formed parallelly in a single focal plane (Dani et al., 2010; Maglione and Sigrist, 2013; Baddeley and Bewersdorf, 2018). STORM and PALM are used with TIRFM in most cases, and parallel formation of PSLM on the glass surface should facilitate image capture processes of STORM or PALM.

Another interesting extension of the culture method on the coated-glass surface is application to studies on presynaptic mechanisms. By coating glass surface with postsynaptic adhesion molecule, formation of presynaptic structure has been achieved (Funahashi et al., 2018). By this method, presynaptic active-zone-like membrane (AZLM) was formed on the glass surface coated with NLG, and single exocytosis event of a synaptic vesicle was visualized. Using an original experimental system, we also demonstrated fast diffusion of a synaptic vesicle protein synaptophysin tagged with SEP on the plasma membrane after membrane fusion of synaptic vesicle membrane, and also distinct distribution patterns of synchronous and asynchronous synaptic-vesicle release locations (Südhof, 2012; Kaeser and Regehr, 2014; Kavalali and Jorgensen, 2014; Maschi and Klyachko, 2017). Thus, the novel imaging preparations enabled by pre- and postsynaptic structure formation directly on the glass surface coated with a synaptic adhesion molecule combined with TIRFM, are expected to shed light on detailed molecular dynamics underlying synaptic transmission and plasticity.

## Author Contributions

TH wrote the manuscript.

## Conflict of Interest Statement

The author declares that the research was conducted in the absence of any commercial or financial relationships that could be construed as a potential conflict of interest.

## Abbreviations

AMPA: α-amino-3-hydroxy-5-methyl-4-isoxazolepropionic acid
AMPAR: AMPA-type glutamate receptor
AZLM: active-zone-like membrane
BSA: Bovine serum albumin
Fc: Fragment crystallizable
LTP: Long-term potentiation
LTD: Long-term depression
LRRTM: Leucine rich repeat transmembrane
NLG: Neuroligin
NMDA: *N*-methyl-D-aspartate
NRX: Neurexin
PALM: Photo-activated localization microscopy
PSLM: Postsynaptic-like membrane
SEP: Super-ecliptic pHluorin
SN: Signal to noise
STORM: Stochastic optical reconstruction microscopy
SynCAM: Synaptic cell adhesion molecule
TIRFM: Total internal reflection fluorescence microscopy.
